# Brachial artery endothelial function is stable across the morning in young men

**DOI:** 10.1186/s12947-015-0036-1

**Published:** 2015-10-05

**Authors:** Saurabh S. Thosar, Chad C. Wiggins, Steven A. Shea, Janet P. Wallace

**Affiliations:** Oregon Institute of Occupational Health Sciences, Oregon Health and Science University, L 606/RJH 1553 A, 97139 Portland, Oregon USA; Department of Kinesiology, School of Public Health, Indiana University Bloomington, Bloomington, Indiana 47405 USA; Department of Public Health and Preventive Medicine, Oregon Health & Science University, Portland, Oregon 97239 USA

**Keywords:** Morning cardiovascular vulnerability, Adverse cardiac events, Flow mediated dilation, Endothelial function, Sleep inertia

## Abstract

**Background:**

The morning hours are associated with increased cardiovascular (CV) risk, and vascular endothelial function (VEF) is a strong predictor of CV disease. A diurnal rhythm in VEF has been established but the morning variation in VEF is not well-documented. Thus, we tested if VEF is impaired across the vulnerable morning period.

**Methods:**

After overnight fasts, eight healthy men (age 26.3 ± 3 yr) underwent assessments of VEF under standardized testing conditions every 2 h from 0700 to 1300 h on two separate days. VEF was estimated following 5 min brachial artery occlusions by hyperemic flow-mediated dilation (FMD).

**Results:**

There was no significant change in FMD or hyperemic shear stimulus across the 6 h vulnerable period on either day, despite changes in physical activity and meals across these periods.

**Conclusion:**

In this healthy group of young men, VEF is stable across the vulnerable morning period when typical behaviors occurred (breakfast and physical activity). Future research should focus on the roles of sleep, physical inactivity during sleep and endogenous circadian rhythm in VEF.

## Background

Adverse cardiovascular (CV) events including myocardial infarction, ventricular arrhythmias and sudden cardiac death occur more frequently early in the morning, especially within 3 h of awakening [1, 2]. It can be argued that these events could be triggered by the stress of awakening from sleep or the initiation of active behaviors across the morning [3] especially in people with established CV risk. In addition, many traditional CV risk markers such as activity-related surges in BP [4], increased pro-thrombotic plasminogen activator inhibitor 1 (PAI-1) [5], cardio-vagal withdrawal [6] and increased alpha-sympathetic drive [6, 7] occur early in the morning and could contribute to this epidemiological finding.

Endothelial function is an important predictor of CV disease [8, 9], and changes in endothelial function can be detected before structural adaptations in the vasculature are detectable by angiography or ultrasound [10]. Endothelial function can be measured as flow mediated dilation (FMD) of the brachial artery during induced hyperemia following release of blood flow occlusion [11]. During FMD measurement, increased blood flow triggers release of nitric oxide (NO) from the vascular endothelium resulting in dilation of the conduit artery [12, 13]. Brachial artery and coronary artery endothelial function are strongly correlated [14] and reduced FMD is a surrogate marker of reduced NO bioavailability and an early hallmark of atherosclerosis [15]. FMD is sensitive to factors including, but not limited to sleep [16], meals, and physical activity [17]. Finally, FMD has been found to exhibit a clear diurnal rhythm with impaired function in the early morning hours [18–25].

Due to its diurnal variation, it has been speculated that lower endothelial function may play a role in the time of adverse CV events [21]. Although numerous studies have documented differences in FMD at various times of the day, to our knowledge, no studies have specifically focused on measuring endothelial function over the period of greatest CV vulnerability (0700–1300 h) while participants perform their normal routine behaviors such as physical activity and eating breakfast. We previously found that FMD does not change between 0800 and 1000 h in healthy individuals while under standardized testing conditions [26]. The goal of the current study was to extend these observations to test if endothelial function is stable for the entire vulnerable morning period (0700–1300 h) in ostensibly healthy individuals while performing standard behaviors such as eating and physical activity.

## Methods

### Study design (Fig. 1)

**Fig. 1:**
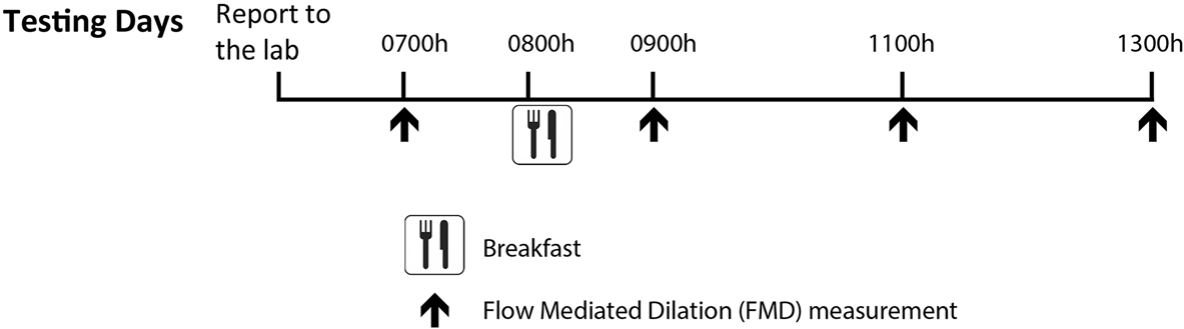
Study design

This study consisted of two screening visits and two experimental visits involving measurement of brachial artery FMD over the course of 6 h on consecutive days as subjects performed their normal daily activities. All procedures for the study were approved by local Institutional Review Board, and the participants gave written informed consent for their participation.

### Participants and screening

Based on self-report, participants were: 1) non-smokers and not using any form of tobacco, 2) free of cardiovascular disease or diabetes, 3) not taking any vasoactive medications or vitamin supplements, and 4) recreationally active (>90 min per week of physical activity, including walking and structured exercise sessions).

Screening visits: At the first visit all experimental procedures were explained to the participants and they were familiarized with the laboratory setting and they provided their written consent. Thereafter, height, weight, and blood pressure were measured using standard procedures, and a medical health history and a questionnaire regarding behavioral habits was completed to screen for any preexisting medical conditions and to determine habitual physical activity levels. Body mass index (BMI) was calculated as kg∙m^−2^. All participants were normotensive (<140/90 mmHg).

### Testing trials

Testing was conducted on two consecutive days. Procedures were identical on both days. Participants were told to not perform any exercise beyond walking for 24 h prior to the trials. Participants wore a fitted Omron HJ-720ITC pedometer [27] immediately upon awakening and throughout the entire testing period each day. Participants arrived at approximately 0630 h at the laboratory after an overnight fast of at least 6 h. Participants were encouraged to slowly walk for approximately 5 min (not more than 10 min) *enroute* to the laboratory so that the FMD measurement would not be confounded by previous prolonged physical inactivity e.g., during the sleep period [28]. After 20 min of supine rest before each measurement, brachial artery FMD was measured at 0700, 0900, 1100, and 1300 h. On both testing days, subjects were fed a standardized low-fat meal consisting of 58 g of Kellogg’s Frosted Flakes®, and 240 mL of skim milk (380 kcal, 76 g carbohydrate, 0 g fat, 18 g protein, 42 g sugar) at 0800 h. A standard low fat breakfast has been shown to not affect brachial artery FMD measurements [29]. Participants were allowed to move freely between measurements on the first day, and on the second day they were told to match the steps accumulated on the first day such that the cumulative steps for each sequential measurement interval were similar on each of the two measurement days.

### Brachial artery flow mediated dilation

Brachial artery FMD was measured according to current guidelines as previously performed in our laboratory [30, 31]. Each measurement was performed in a quiet and climate controlled (22–25 °C) room. An automatic blood pressure cuff (E-20 rapid cuff inflator; D.E. Hokanson, Bellevue, Wash., USA) was placed on the right forearm. Images of the brachial artery were obtained with a 2-D high-resolution ultrasound system (Terason t3000, Teratech Corp., Burlington, Mass., USA) using a 5- to 12-MHz multi-frequency linear-array transducer. Once satisfactory images of near and far arterial walls were obtained, the transducer was secured and stabilized in a stereotactic clamp and landmarks were made on the participant’s skin to ensure similar placement of the transducer for subsequent assessments. In addition to imaging the arterial dimensions, Doppler ultrasound was used to concurrently measure brachial artery blood velocity. Doppler flow signals were corrected to an insonation angle of 60° and the sample volume was placed in the middle of the artery. Vessel images and Doppler measurements of blood velocity were continuously recorded for 1 min at baseline prior to cuff inflation. The automatic blood pressure cuff was then rapidly inflated to 250 mmHg and this pressure was maintained for 5 min. Diameter and blood velocity recordings resumed 30 s prior to cuff deflation and continued for 3 min after deflation. Ultrasound images were continuously recorded at 5 frames · s^−1^ with Camtasia (TechSmith, Okemos, Mich., USA), and stored as .avi files [32]. This same procedure was repeated at each measurement interval.

Arterial diameters and blood velocities: Off-line analyses of arterial diameters were performed using automated edge-detection software (Brachial Analyzer, Medical Imaging Applications LLC, Coralville, IA, USA) as previously described [33]. This software enables determination of a region of interest in which the near and far vessel walls are clearest. The vessel wall borders are then detected using an optimal graph search-based segmentation that uses a combination of pixel density and image gradient as an objective function. All analyzed images were reviewed by an investigator and edited as needed to ensure that diameter measures were determined from the intima-lumen interface at the near and far vessel wall. Blood velocities were determined using custom written software selecting a region of interest that surrounded the Doppler wave [32]. The velocity–time integral was used to calculate the mean blood velocity. The peak dilation after cuff deflation was determined as the highest 3 s moving average and recorded as a percentage change from baseline diameter (FMD %). Shear rate was calculated as the area under the curve from the time of deflation up until peak diameter (SRauc). For consistency, all measurements were performed by the same investigator (SST) who was blinded to the participant identity and time code of each image file.

### Statistical analysis

Descriptive statistics were used to summarize subject characteristics. Repeated measures ANOVAs were conducted to test for differences in baseline diameter (BD) and SRauc across time. A mixed model ANOVA was then conducted on FMD as the dependent variable with time and day as a fixed factor, and step count and BD as covariates. Finally, bivariate correlations were tested between FMD and step count between FMD measurements. All values are expressed as the mean ± SD unless otherwise specified. The *a priori* alpha level for statistical significance was set at 0.05. To better understand the uncertainty in measurements of BD and FMD, we also estimated variance components associated with subject, experimental replication (i.e., day), and time.

## Results

**Fig. 2:**
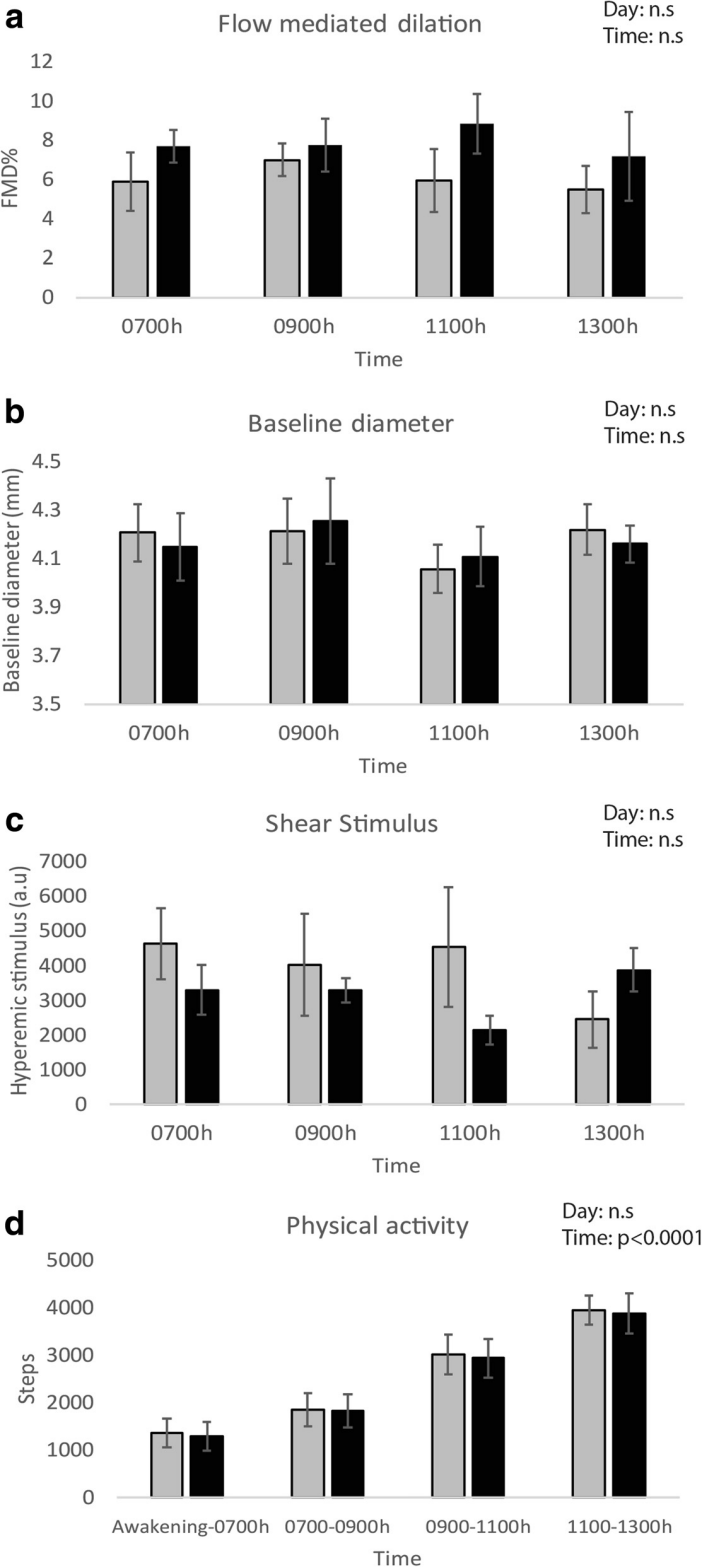
Graphical representation of **a** FMD, **b** Baseline diameter, **c** Hyperemic shear and **d** Physical activity at each measurement on both days. *Gray bars* represent Day 1, *Black bars* represent Day 2. *Error bars* represent standard error

We recruited and tested eight subjects who comprised a homogenous group of ostensibly healthy, active young men (Table 1). All results are presented in Fig. 2

**Table 1:** Subject demographics

Variable	Value
N	8
Age (yrs.)	26.3 ± 3
BMI (kg.m^2^)	22.49 ± 1.7
Systolic blood pressure (mm Hg)	114 ± 6
Diastolic blood pressure (mm Hg)	76 ± 6

Data are presented as mean ± SD

.

Baseline diameter (BD): There was no significant difference in BD between days (F_1,49_ < 0.19, *p* = 0.66) and across time (F_3,49_ = 1.57, *p* = 0.16), and no interaction between day and time (*p* = 0.79).

SRauc: There was no significant difference in SRauc between days (F_1,26_ = 3.90, *p* = 0.06) and across time (F_3,26_ = 0.16, *p* = 0.92), and no interaction between day and time (*p* = 0.88).

FMD: There was no significant difference in FMD between days (F_1,45_ = 3.4, *p* = 0.07) and across time (F_3,45_ = 0.19, *p* = 0.90), and no interaction between day and time (*p* = 0.85) even while controlling for BD and inter subject variability.

Activity: There was no significant difference in step counts between days (F_1,52_ = 0.16, *p* = 0.69). There was a systematic difference in step counts across the morning (F_3,52_ = 57.81, *p* < 0.0001), and this pattern was similar on both days (and no interaction between day and time (*p* = 0.99)). There was no significant correlation between FMD and step count across the morning period (*r* = −0.1597, *p* = 0.23).

To better understand the uncertainty in measurements of BD and FMD, we estimated variance components associated with subject, day and time. In this model there is only a single overall mean response, either for BD or FMD, with individual observations resulting from (random) perturbations from the mean according to the subject, the day, or time within a day (Table 2). The magnitude of the standard deviations and their confidence intervals are comparable to previously published reports involving interventions using low intensity physical activity [32] and vitamin C [34] over time in a single day.

**Table 2:** Variability in SD of BD and FMD stemming from subjects, measurements between days and measurements across time

Source of variability	Baseline diameter (mm) SD (95 % CI)	FMD% SD (95 % CI)
Subject	0.271 (0.153, 0.479)	2.20 (1.03, 4.70)
Day	0.091 (0.023, 0.398)	2.16 (1.06, 4.42)
Time	0.174 (0.124, 0.244)	2.75 (2.00, 3.80)

## Discussion

The goal of this study was to test if FMD is stable during across the morning hours which are associated with increased incidence of adverse CV events even in ostensibly healthy individuals [1]. Thus, in this pilot study, as the first step towards understanding the contribution of endothelial function to adverse CV events, before studying participants with increased CV risk, we chose to study healthy individuals. Baseline measurements were made after at least 6 h of overnight fasting, and thereafter across the measurement period participants ate a standardized breakfast and performed light intensity physical activity (walking) to simulate a natural daily activity and food pattern. Under these conditions, we discovered that FMD is stable during the morning period in this group of healthy men. Our findings extend our previous finding that FMD was stable across the 0800–1000 h period [26]. Our findings support previous findings by Kawano et al. [19] who reported that FMD is quite stable across the morning despite strictly controlled posture between measurements. Bau et al. [35] also found little change in FMD across the morning but they do not report the amount of activity or meal composition between measurements. Bau and colleagues [35] did find that brachial artery baseline diameter was lowest at 0700 h compared to afternoon measurements. We did not find any significant difference in our baseline diameters between 0700 and 1300 h. Assuming that increased alpha sympathetic activity increases across this same morning period [7] our finding would suggest that alpha sympathetic activity does not affect brachial artery baseline diameters in our group. Because we tested participants on two separate days and found the same result our study also establishes reproducibility within our measurements and strengthens the argument that in young healthy men performing normal daily activities, FMD is largely stable during the morning hours.

Our findings are discordant with the findings of Otto and colleagues [18] who discovered that endothelial function was significantly higher at 11 AM as compared to 6 AM. In their well-controlled study, they measured FMD before sleep, immediately upon awakening and 5 h after awakening. A main difference between our studies is that in Otto el al’s. participants were awakened at 0600 h whereas our participants woke on their own volition and travelled to the laboratory, such that we had no measurements immediately following sleep. It is interesting to note that Otto et al’s mean FMD values at 2100 h and at 1100 h are approximately 7.5 % and our mean FMD across all time points is very similar 6.9 %. Therefore, the fact that Otto and colleagues found FMD immediately at awakening to be greatly diminished (4.4 %) strongly suggests that the effect of sleep and/or the physical inactivity that accompanies sleep [28] reduces endothelial function across the night and that this persists immediately upon awakening, akin to a sleep inertia [16] of vascular function. However, FMD appears to quickly improve within about 2 h of awakening. There is also a possibility of an endogenous circadian rhythm in FMD causing FMD to increase in the 2 h period following waking. It is thus essential to separate out the effects of sleep, physical inactivity and endogenous circadian rhythm in FMD to truly understand the predictive value of FMD in morning CV events.

Our study adds to the current body of knowledge in other important ways. For instance, we also measured the hyperemic stimulus after cuff release (SRauc) which has been shown to influence FMD and which is also a measure of microvascular function [36]. Previously, it was unknown if SRauc changes and/or affects FMD measurements during the early morning hours. Our study suggests that in healthy young men SRauc is largely stable across the morning hours. Our study is also important for scientists who study the effects of high fat or high sugar meals on endothelial function [17]. Most of these studies are conducted in a fasting state in the morning and it is sometimes difficult to differentiate the effects of high fat/sugar meals versus the plain effect of time. From our study, it is clear that in this population, any changes in FMD due to a high fat/sugar meal, are likely to be due to the experiment and not time into the protocol.

Mechanisms: There are various mechanisms which may affect endothelial function in the early morning hours, and which may play a role in adverse CV events in the morning. For instance, it is known that plasminogen activator inhibitor −1, which adversely affects endothelial function has a diurnal and a circadian rhythm that peaks in the morning [5, 37]. In addition, there is an increase in sympathetic tone in the early morning hours [7] which may adversely affect endothelial function [38]. However, our data suggest that the cumulative effect of various mechanistic variables is largely constant in the early morning hours, in young healthy individuals.

### Limitations

We did not keep physical activity constant between measurements on each trial day, which is a concern because minimal intensity physical activity affects FMD [32]. However, we had participants control their activity levels on Day 2 to match those on Day 1. And since there were changes in activity across the day on each day, but no changes in FMD, it seems likely that there was an increase in VEF between awakening and our first measurement time which was associated with the lowest level of accumulated steps (Fig. 2), which would mean that even a minimal level of activity may be sufficient to increase endothelial function in the morning. Second, our study may be limited because of relatively small sample size. However, this limitation is partly compensated for by repeating all measurements in each participant on two separate days. Ours was an initial pilot study and future research using larger sample sizes including different population groups is necessary before generalizing our findings. We have also provided the reader the variability and confidence intervals in our data (Table 2). The magnitude of the standard deviations and their confidence intervals are comparable to previously published reports involving interventions using low intensity physical activity [32] and vitamin C [34] over time in a single day. Third, we did not study biomarkers, heart rate, blood pressure and smooth muscle dilation. This was an initial study to test if FMD is stable across the vulnerable morning period. Whereas biomarkers and other physiological measurements will have allowed us to see if the mechanisms controlling, we also found that FMD is largely stable across the morning. Finally, we did not control the amount of participants’ sleep. Participants were informed that they should sleep for 8 h and awaken at the same times for both trials, but sleep was not monitored. It is possible that differences in sleep time and quality may have different effects on FMD. Conversely, sleeping in a laboratory may have adversely affected sleep and interfered with the validity of measurements.

## Conclusion

In healthy young men, vascular endothelial function is stable during the morning between of 0700 and 1300 h, but our results need validation using large sample sizes before they can be generalized. In addition future studies should separately evaluate the effects of physical inactivity, prolonged sleep and endogenous circadian system on endothelial function in the morning, in healthy people and those with chronic diseases including CV disease.
